# Inhibition of S6K1 accounts partially for the anti-inflammatory effects of the arginase inhibitor L-norvaline

**DOI:** 10.1186/1471-2261-9-12

**Published:** 2009-03-13

**Authors:** Xiu-Fen Ming, Angana Gupta Rajapakse, João Miguel Carvas, Jean Ruffieux, Zhihong Yang

**Affiliations:** 1 Vascular Biology, Department of Medicine, Division of Physiology, University of Fribourg, Switzerland

## Abstract

**Background:**

Pharmacological inhibition of endothelial arginase-II has been shown to improve endothelial nitric oxide synthase (eNOS) function and reduce atherogenesis in animal models. We investigated whether the endothelial arginase II is involved in inflammatory responses in endothelial cells.

**Methods:**

Human endothelial cells were isolated from umbilical veins and stimulated with TNFα (10 ng/ml) for 4 hours. Endothelial expression of the inflammatory molecules i.e. vascular cell adhesion molecule-1 (VCAM-1), intercellular adhesion molecule-1 (ICAM-1), and E-selectin were assessed by immunoblotting.

**Results:**

The induction of the expression of endothelial VCAM-1, ICAM-1 and E-selectin by TNFα was concentration-dependently reduced by incubation of the endothelial cells with the arginase inhibitor L-norvaline. However, inhibition of arginase by another arginase inhibitor S-(2-boronoethyl)-L-cysteine (BEC) had no effects. To confirm the role of arginase-II (the prominent isoform expressed in HUVECs) in the inflammatory responses, adenoviral mediated siRNA silencing of arginase-II knocked down the arginase II protein level, but did not inhibit the up-regulation of the adhesion molecules. Moreover, the inhibitory effect of L-norvaline was not reversed by the NOS inhibitor L-NAME and L-norvaline did not interfere with TNFα-induced activation of NF-κB, JNK, p38mapk, while it inhibited p70s6k (S6K1) activity. Silencing S6K1 prevented up-regulation of E-selectin, but not that of VCAM-1 or ICAM-1 induced by TNFα.

**Conclusion:**

The arginase inhibitor L-norvaline exhibits anti-inflammatory effects independently of inhibition of arginase in human endothelial cells. The anti-inflammatory properties of L-norvaline are partially attributable to its ability to inhibit S6K1.

## Background

The vessel wall inflammation constitutes a major factor in atherogenesis [1,2]. The initial step of atherosclerosis is inflammatory cell invasion into the intima upon the onset of inflammation. This process is predominantly mediated by the interaction of complementary cellular adhesion molecules on the endothelium and the inflammatory cells. The endothelial adhesion molecules, including vascular cell adhesion molecule-1 (VCAM-1), intercellular adhesion molecule-1 (ICAM-1) and E-selectin, are expressed on the vascular endothelium when the endothelial cells (ECs) undergo inflammatory activation in response to cytokines such as TNFα [3]. E-selectin is involved in the rolling and tethering of leukocytes on the vascular wall, whereas VCAM-1 and ICAM-1 induce firm adhesion of inflammatory cells at the vascular surface [3]. Several lines of evidence support a crucial role of endothelial adhesion molecules in the development of atherosclerosis and plaque instability. Expression of VCAM-1, ICAM-1 and E-selectin has been observed in ECs of human atherosclerotic lesions [4,5]. Moreover, mice genetically deficient in any of these endothelial adhesion molecules have reduced atherosclerotic lesion formation in atherosclerosis-prone ApoE-/- or LDLR-/- mice [6-10].

The endothelium-derived nitric oxide (NO), an important vasoprotective factor, is produced from the substrate L-arginine by endothelial NO synthase (eNOS) [11]. In addition to the functions in regulation of vascular tone and inhibition of platelet aggregation [12], NO has also been shown to inhibit leukocyte adhesion to the endothelium [13,14], which might be partially attributable to the inhibition of endothelial VCAM-1 and ICAM-1 expression [15,16]. Recent research work provides accumulating evidence for the involvement of arginase in decreased endothelial NO production through competition with eNOS for the substrate L-arginine [17]. There is increasing number of studies showing that enhanced arginase gene expression and/or activity contribute to endothelial dysfunction in various cardiovascular disorders including atherosclerosis [18,19]. Most recently, it was reported that in vivo pharmacological treatment of ApoE-/- mice with arginase inhibitors improves endothelium-dependent relaxations and reduces atherosclerotic plaque lesion formation [19], implicating the potential role of endothelial arginase in pathogenesis of vascular diseases. It has however, not been confirmed whether the beneficial effects of arginase inhibitors are indeed attributable to the inhibition of arginase, in another word, whether the endothelial arginase is indeed involved in inflammatory responses. In the present study, we elucidated the pharmacological effects of arginase inhibitors on endothelial inflammations and the role of arginase in endothelial inflammation was then clarified by small interfering RNA (siRNA)-based specific knocking down of the enzyme in the cells.

## Methods

### Materials

Reagents were purchased from the following sources: recombinant human TNFα was purchased from Brunschwig (Basel, Switzerland); S-12-bromoethyl-L-cystine-HCl (BEC) was from Calbiochem; L-norvaline and anti-tubulin monoclonal antibody were from Sigma (Buchs, Switzerland). Both BEC and L-norvaline are dissolved in distilled water and further diluted in culture medium. Anti-VCAM-1, -ICAM-1 and -E-selectin antibodies were from Santa Cruz (Nunningen, Switzerland); anti-IκB, -phospho-IκB-S32, -phospho-c-jun-S63, -phospho-CREB-S133, and -phospho-S6-S235/236 were from Cell Signaling (Allschwil, Switzerland); rabbit anti-S6K1 was from Dr. G. Thomas (Genome Research Institute, University of Cincinnati, USA) [20]; Endothelial cell growth supplement (ECGS) pack was from Promocell GmbH (Allschwil, Switzerland); all of the cell culture medium and materials were purchased from Gibco BRL (Basel, Switzerland).

### Cultivation of human umbilical vein endothelial cells (HUVECs)

Endothelial cells were isolated from human umbilical veins and cultivated as described [21]. Cells of 1^st ^to 4^th ^passages were used.

### Generation of recombinant adenoviral (rAd) expressing short hairpin RNA (shRNA)

Generation of recombinant adenovirus expressing shRNA targeting human arginase II (ARG II) or p70s6k (S6K1) [22] driven by the U6 promoter (rAd/U6-ARGII^shRNA ^or -S6K1^shRNA^, respectively) was carried out with the Gateway Technology (Invitrogen life Technologies). The sequence below (only the sense strand is shown) was first cloned into the Gateway pENTR/U6 Vector. A LR recombination reaction between the entry clone pENTR/U6-ARGII^shRNA ^or -S6K1^shRNA ^and a Gateway destination vector pAd/BLOCK-iT-DEST was then performed to obtain a recombinant adenoviral expression clone pAd/U6-ARGII^shRNA ^or -S6K1^shRNA^, respectively. The resultant recombinant virusmids were digested with PacI and transfected into E1-complementing packaging cells (HER911) to generate recombinant adenoviruses. The primary crude lysates of the recombinant adenoviruses were prepared as viral stocks and used to infect HER911 cells to scale up the viral preparation. The viral titer was determined by plaque assay. The control recombinant adenoviruses expressing shRNA targeting LacZ was from Invitrogen life Technologies. In boldface are the targeting DNA sequences.

Arginase II targeting sequence:

a. CACC**GCAGTAGATGTGATTGCTT**CGAAAAGCAATCACATCTACTGC;

b. CACC**GCATATTGTCTATGACCAACT**CGAAAGTTGGTCATAGACAATATGC;

c. CACC**GCGAGTGCATTCCATCCTGAA**CGAATTCAGGATGGAATGCACTCGC.

S6K1 targeting sequence: CACC**GGACATGGCAGGAGTGTTTGA**CGAATCAAACACTCCTGCCATGTCC.

### Adenoviral transduction of HUVEC

HUVEC were seeded at a density of 2 × 10^5^/6 cm dish in complete RPMI-1640 supplemented with 5% FCS and ECGS. Two days later, cells were transduced with the recombinant adenovirus at titers of 100 MOI and cultured in complete medium for 3–4 days. The cells were then serum-starved in 0.2% FCS RPMI-1640 for 24 hours before treatment and extraction on days 4 of post-transduction (4 d p.t).

### Expression of the adhesion molecules VCAM-1, ICAM-1, E-selectin

Cell lysate preparation, SDS-PAGE, transfer of SDS gels to an Immobilon-P membrane (Millipore) were performed as previously described [21]. The resultant membrane was first incubated with the corresponding primary antibody at room temperature for 2 – 3 hours with gentle agitation after blocking with 5% skimmed milk. The blot was then further incubated with a corresponding anti-mouse (Alexa fluor 680 conjugated) or anti-rabbit (IRDye 800 conjugated) secondary antibodies and the signals were visualized using Odyssey Infrared Imaging System (LI-COR Biosciences). Quantification of the signals was performed using the Odyssey Application Software 1.2.

### Activation of NF-κB, JNK, p38mapk, and S6K1

Activation of NF-κB, JNK, p38mapk and S6K1 pathways was assessed by immunoblotting of phospho-IκB-S32/total IκB, phospho-c-jun-S63, phospho-CREB-S133, and phospho-S6-S235/236, respectively.

### Statistics

Data are given as mean ± SEM (standard error of mean). In all experiments, n equals the number of individual experiments. The ANOVA with Bonferroni's post-test was used for statistical analysis. Differences in mean values were considered statistically significant at p < 0.05.

## Results

### L-norvaline inhibits TNFα-induced endothelial inflammation

**Figure 1:**
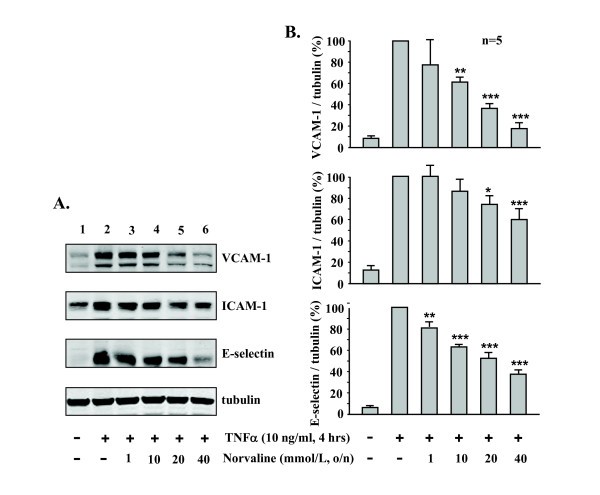
**Effect of L-norvaline on TNFα-induced expression of VCAM-1, ICAM-1, and E-selectin**. (A) Stimulation of HUVECs with TNFα (10 ng/ml, 4 hours) increased expression of VCAM-1, ICAM-1, and E-selectin, which was significantly reduced by L-norvaline in a concentration-dependent manner. Tubulin in bottom panel served as loading control. (B) Quantification of the signals shown in panel A. Representative data from five independent experiments are reported as mean ± SEM. Values are given as percentage relative to stimulation with TNFα. *p < 0.05, **p < 0.01, ***p < 0.001 vs. stimulation with TNFα. o/n = overnight.

Stimulation of the endothelial cells with TNFα (10 ng/ml) for 4 hours strongly enhanced the expression of the examined inflammatory adhesion molecules, i.e. VCAM-1, ICAM-1, E-selectin as assessed by immunoblotting analysis (Fig. 1, **lane 2**, n = 5). This effect of TNFα was concentration-dependently prevented by pre-incubation of the cells with the arginase inhibitor L-norvaline (1 to 40 mmol/L) (Fig. 1, lanes 3–6, n = 5, ***p < 0.001, **p < 0.01, *p < 0.05 vs. TNFα alone).

### L-norvaline inhibits endothelial inflammation independently of NOS activity

**Figure 2:**
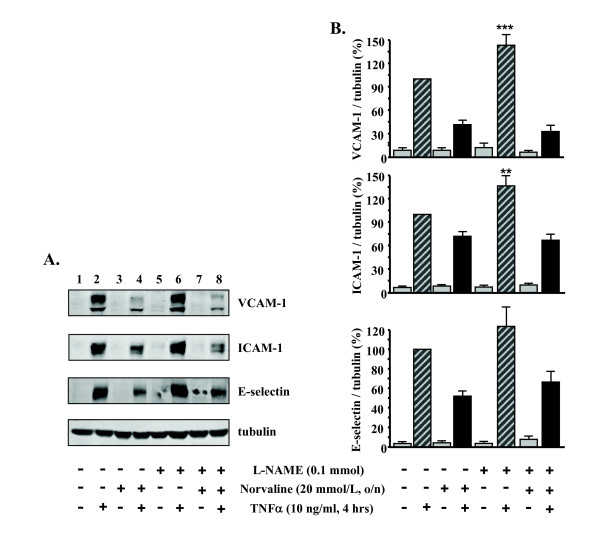
**L-norvaline-mediated inhibition of VCAM-1, ICAM-1, and E-selectin: independence of NOS activity**. (A) The effects of L-norvaline on TNFα-induced expression of VCAM-1, ICAM-1, and E-selectin could not be reversed by the NOS inhibitor L-NAME (100 μmol/L). Tubulin in bottom panel served as loading control. Shown are representative blots from five independent experiments. (B) Quantification of the signals shown in panel A. Values are given as percentage relative to stimulation with TNFα in the absence of L-NAME. **p < 0.01, ***p < 0.001 vs. stimulation with TNFα in the absence of L-NAME.

To examine whether the inhibitory effects of L-norvaline on expression of VCAM-1, ICAM-1, E-selectin are through increased NOS activity, the endothelial cells were pre-incubated with L-NAME (0.1 mmol/L) for 1 hour (to inhibit NOS activity in the cells [18]) before treatment with L-norvaline (20 mmol/L) followed by stimulation with TNFα for 4 hours. As shown in Fig. 2, L-NAME did not reverse the effects of L-norvaline (Fig. 2, **compare lane 8 vs. lane 4**), although L-NAME significantly augmented the TNFα-induced effect on the VCAM-1 and ICAM-1 expression (Fig. 2, lane 6 vs. lane 2, n = 5, ***p < 0.001, **p < 0.01 vs. TNFα alone).

### L-norvaline inhibits endothelial inflammation independently of arginase

**Figure 3:**
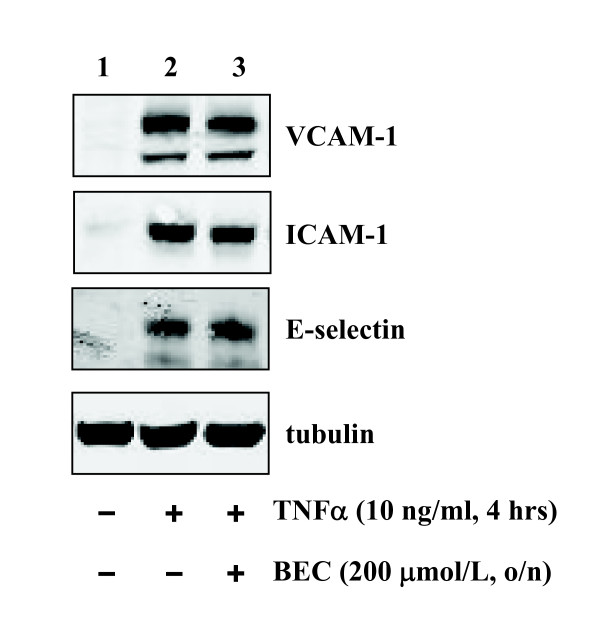
**Role of the arginase inhibitor BEC on TNFα-induced expression of VCAM-1, ICAM-1, and E-selectin**. Stimulation of the cells with TNFα (10 ng/ml, 4 hours) increased inflammatory response which was not affected by the arginase inhibitor BEC (200 μmol/L, o/n). Shown are representative blots from five independent experiments.

**Figure 4:**
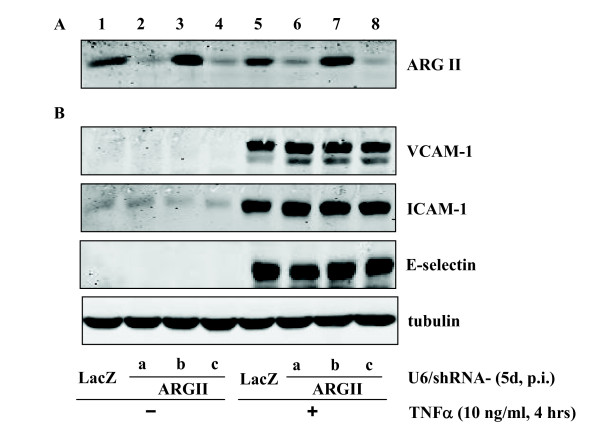
**Role of arginase II silencing on TNFα-induced expression of VCAM-1, ICAM-1, and E-selectin**. HUVECs were transduced with recombinant adenovirus expressing shRNA against LacZ as control (lanes 1 and 5) or arginase II (lanes 2–4 and 6 – 8). On day 4 of post transduction, cells were serum-starved for 20 h followed by stimulation with TNFα (10 ng/ml, 4 hours) and extracted. The knocking down effects of various shRNA targeting sequences were assessed by immunoblotting as shown in panel A. Panel B shows the effects on expression of VCAM-1, ICAM-1, and E-selectin. Shown are representative blots from five independent experiments.

Next we investigated whether the anti-inflammatory effects of L-norvaline are dependent on arginase activity. For this purpose, the endothelial cells were either pre-treated with another arginase inhibitor BEC (Fig. 3) or transduced with recombinant adenovirus expressing shRNA targeting arginase-II (Fig. 4), the principle arginase isoform in HUVECs [18]. Our experiments showed that BEC at the concentration of 200 μmol/L exerted inhibitory effects on arginase activity (51.9 ± 7.0% inhibition) which was comparable to that of 20 mmol/L L-norvaline (52.4 ± 3.9% inhibition, n = 4). However, in contrast to L-norvaline, BEC was unable to affect TNFα-induced expression of any of the inflammatory molecules (Fig. 3, n = 5). Similarly, knocking down arginase-II by the two different shRNAs i.e. shRNA-ARG IIa and c (shRNA-ARG IIb had no effect and served as a 2^nd ^control in addition to shRNA-LacZ) also had no effects (Fig. 4, lanes 6 and 8 vs. lanes 5 and 7, n = 5).

### L-norvaline inhibits S6K1

**Figure 5:**
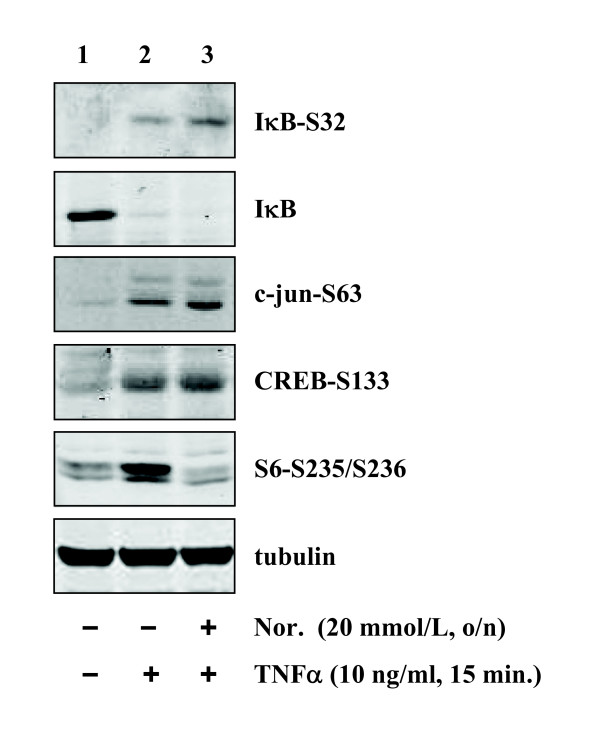
**Effects of L-norvaline on TNFα-induced activation of various signaling pathways**. After pre-treatment of the cells with L-norvaline (Nor. 20 mmol/L), the cells were stimulated with TNFα (10 ng/ml, 15 min) as indicated. The extracts were subjected to immunoblotting to examine the activation of NF-κB by monitoring degradation and phosphorylation of IκB. The activation of JNK, p38mapk and S6K1 pathways were assessed by monitoring phosphorylation of their substrate c-jun, CREB and S6, respectively. Shown are representative blots from four independent experiments.

Given the above observations, we then tested whether the anti-inflammatory effects of L-norvaline are attributable to its ability to interfere with signaling pathway(s) involved in expression of the inflammatory molecules. Upon stimulation with TNFα (10 ng/ml, 15 minutes), NF-κB, JNK, p38mapk and S6K1 pathways were activated, as monitored by phosphorylation and degradation of IκB for NF-κB activation, and phosphorylation of the substrate of JNK, p38mapk and S6K1: c-jun, CREB and S6, respectively (Fig. 5, lane 2). Pre-treatment of the cells with L-norvaline (20 mmol/L) did not significantly affect the activation of NF-κB, JNK or p38mapk, but remarkably inhibited S6K1 activation (Fig. 5, lane 3).

### Role of S6K1 in up-regulation of VCAM-1, ICAM-1, and E-selectin

**Figure 6:**
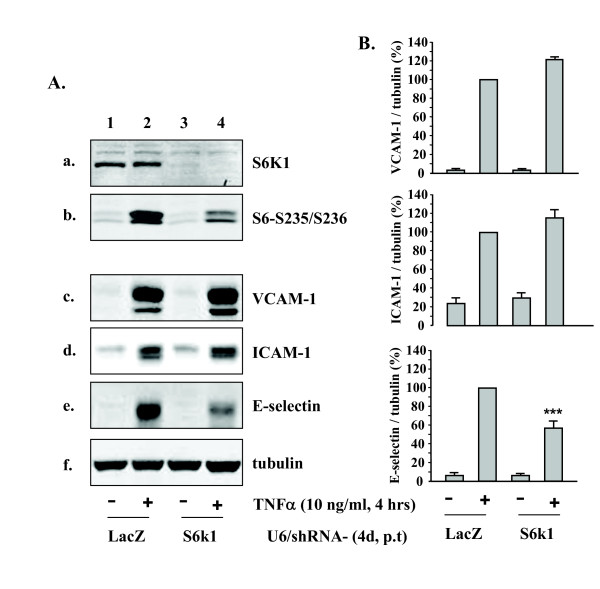
**Effects of silencing S6K1 on expression of VCAM-1, ICAM-1, and E-selectin**. HUVECs were transduced with recombinant adenovirus expressing shRNA against LacZ as control (lanes 1 and 2) or S6K1 (lanes 3 and 4). On day 4 of post transduction, cells were serum-starved for 20 h followed by stimulation with TNFα (10 ng/ml) for 4 hours and extracted. (A) The knocking-down effects of shRNA targeting S6K1 were assessed by immunoblotting for the expression of S6K1 (panel a) and for phosphorylation of its substrate S6 (panel b). Panels c to e reveal the effects on expression of VCAM-1, ICAM-1, and E-selectin. Shown are representative blots from five independent experiments. (B) Quantification of the signals in (A) (panels c – e). All blots were normalized to tubulin expression. Representative data from five independent experiments are reported as mean ± SEM. Values are given as percentage relative to stimulation with TNFα in the control LacZ-shRNA-transduced cells. ***p < 0.001 vs. stimulation with TNFα in the control LacZ-shRNA-transduced cells.

The role of S6K1 in up-regulation of VCAM-1, ICAM-1, and E-selectin was then further investigated by recombinant adenovirus-mediated RNA interference (RNAi). In parallel to the abrogated expression of S6K1 (Fig. 6A, panel a), cells transduced with the rAd-shRNA against S6K1 exhibited significantly decreased kinase activity upon stimulation with TNFα (10 ng/ml, 4 hours), as measured by phosphorylation of its substrate S6 at serine235/serine236 (Fig. 6A, panel b). In the cells transduced with control viral vector expressing LacZ-shRNA, TNFα (10 ng/ml, 4 hours) induced up-regulation of VCAM-1, ICAM-1, and E-selectin (Fig. 6A, **panels c to e**, lane 2; n = 5, p < 0.001 vs. LacZ alone lane 1), silencing S6K1 significantly reduced TNFα-induced up-regulation of E-selectin, but not that of VCAM-1 and ICAM-1 (Fig. 6B, lane 4; n = 5, p < 0.001 vs. LacZ + TNFα, lane 2).

## Discussion

Emerging evidence suggests that increased endothelial arginase activity decreases L-arginine availability for eNOS to produce NO under various pathological conditions [17]. The arginase inhibitor L-norvaline has been previously shown to improve endothelial NO release via inhibition of arginase [18]. Treatment of ApoE-/- mice with arginase inhibitors has been reported to improve endothelial function and reduce plaque formation [19]. It is however, not clear whether arginase is indeed involved in vascular inflammation responses, a crucial mechanism in atherogenesis. The present study further investigated whether arginase is involved in the modulation of endothelial inflammatory responses and whether this is dependent on NOS activity in endothelial cells. In cultured human endothelial cells, we demonstrate that the arginase inhibitor L-norvaline exerts anti-inflammatory effect at the same concentration ranges which exhibit arginase inhibitory activity [18]. It inhibited endothelial expression of VCAM-1, ICAM-1, E-selectin stimulated by TNFα. Although inhibition of NOS activity in the endothelial cells with L-NAME [18] could significantly augment TNFα-induced expression of VCAM-1 and ICAM-1, it was unable to reverse the anti-inflammatory effects of L-norvaline. The results implicate that basal level of NO exerts a weak but significant anti-inflammatory effects, which is consistent with the previous findings [15,16]. The results with L-NAME suggest that L-norvaline inhibits endothelial inflammation via a mechanism(s) independently of NO production and/or arginase. To further confirm the role of arginase-II, the prominent isoform expressed in HUVECs [18] in the inflammatory responses, another arginase inhibitor BEC and adenoviral mediated siRNA silencing of arginase-II were employed. Indeed, pharmacological inhibition of arginase activity with BEC at the concentration (200 μmol/L) exerts similar inhibition of arginase activity (51.9 ± 7.0% inhibition) as 20 mmol/L L-norvaline (52.4 ± 3.9% inhibition); however, it did not show any effects on TNFα-induced expression of the adhesion molecules. Moreover, efficient knocking down of arginase-II with two different targeting sequences (shRNA-ARGIIa and -ARGIIc) did not affect endothelial expression of the inflammatory molecules stimulated by TNFα either. Both pharmacological and specific siRNA silencing approaches therefore reinforce our conclusion that L-norvaline exerts anti-inflammatory effects in endothelial cells independently of arginase and NOS activity.

Another important finding of this report is the role of S6K1 in up-regulation of E-selectin by TNFα. In an effort to analyse the possible underlying mechanisms of L-norvaline-mediated inhibition of endothelial inflammatory responses, we investigated whether the anti-inflammatory effects of L-norvaline are attributable to its ability to interfere with signaling pathway(s) that could regulate the expression of adhesion molecules. Various signalling pathways such as NF-κB, JNK, p38mapk have been shown to be involved in the up-regulation of VCAM-1, ICAM-1, E-selectin [23-26]. These mechanisms were however, not affected by L-norvaline, demonstrating that the compound inhibits endothelial inflammation independently of the signalling mechanisms. Interestingly, we found that TNFα-induced S6K1 activation was strongly inhibited by L-norvaline, indicating that the compound may inhibit endothelial inflammation via inhibition of S6K1. To confirm this hypothesis, we took the siRNA approach to knock-down the expression and activity of S6K1. Remarkably, silencing experiments revealed that suppressing S6K1 expression and the enzymatic activity inhibited the expression of E-selectin, but not that of VCAM-1 or ICAM-1 in response to TNFα. The results demonstrate that the anti-inflammatory properties of L-norvaline are partially attributable to its ability to inhibit S6K1. This is the first study showing that S6K1 plays an important role in the expression of endothelial inflammatory molecule E-selectin. The mechanisms that are involved in L-norvaline-mediated inhibition of endothelial VCAM-1 and ICAM-1 expression remain to be investigated in the future study.

## Conclusion

In summary, our results demonstrate that the arginase inhibitor L-norvaline exhibits anti-inflammatory properties independently of its effect on inhibition of arginase activity and increased NO production. The anti-inflammatory effects are partially attributable to its ability to inhibit S6K1. This effect of L-norvaline has to be taken into account, when the drug is used to study functions of arginase or NO production in biological systems. The results of our study also implicate that L-norvaline or its derivatives may be useful drugs to treat inflammatory responses in cardiovascular system and to prevent cardiovascular diseases.

## Abbreviations

ARG II: arginase II; BEC: S-12-bromoethyl-L-cystine-HCl; ECs: endothelial cells; ECGS: endothelial cell growth supplement; eNOS: endothelial nitric oxide synthase; HUVEC: human umbilical vein endothelial cells; ICAM-1: intercellular adhesion molecule-1; JNK: c-jun N-terminal kinase; NO: nitric oxide; o/n: overnight; p.t.: post transduction; rAd: recombinant adenovirus; SEM: standard error of mean; shRNA: short hairpin RNA; siRNA: small interfering RNA; TNFα: tumor necrosis factor alpha; VCAM-1: vascular cell adhesion molecule-1.

## Competing interests

The authors declare that they have no competing interests.

## Authors' contributions

XFM conceived the study, performed Western blot analysis, constructed rAd/U6-shRNAs and drafted the manuscript. AGR and JMC assisted in adenoviral transduction and Western blot analysis. JR assisted in cultivation of HUVECs. ZY conceived and coordinated the study, and drafted the manuscript. All authors read and approved the final manuscript.

## Pre-publication history

The pre-publication history for this paper can be accessed here:


